# Polarization dependent light propagation in $\textrm{WTe}_2$ multilayer structure

**DOI:** 10.1038/s41598-023-40460-7

**Published:** 2023-08-14

**Authors:** S. Oskoui Abdol, S. Shojaei, B. Abdollahipour

**Affiliations:** 1 Faculty of Physics, University of Tabriz, Tabriz, 51666-16471 Iran; 2 Research Institute for Applied Physics and Astronomy (RIAPA), University of Tabriz, Tabriz, 51655-163 Iran

**Keywords:** Optical materials and structures, Physics, Condensed-matter physics, Electronics, photonics and device physics

## Abstract

$\textrm{WTe}_2$ is one of the exciting and outstanding semimetallic members of TMDCs, which has attracted immense attention for manipulating light propagation due to its inherent optical anisotropy and hyperbolic characteristic in the infrared frequency range. We investigate the dependence of the reflectance and transmittance of structures with a single and double $\textrm{WTe}_2$ thin film in terms of frequency and polarization angle of the incident wave. We find rich behaviors in the optical response of these structures due to their anisotropic permittivity tensors. Furthermore, we analyze the polarization state of transmitted and reflected waves through these structures. We demonstrate that these structures provide the ability to achieve desired polarization rotation for outgoing waves by tuning the frequency and polarization angle of the incident wave with respect to the principal axes of $\textrm{WTe}_2$ thin film. In particular, we elucidate the essential relevance of the optical response and polarization rotation of the double thin film structure to the in-plain twist angle of $\textrm{WTe}_2$ thin films. We explain that this structure permits comprehensive control of the polarization rotation of the outgoing waves by adjusting the twist angle of thin films. The proposed structure can be employed as an efficient light manipulator with the aim of application in communication, imaging, and information processing.

## Introduction

Recently, transition-metal dichalcogenides (TMDCs) have attracted considerable attention in materials research due to their outstanding features in the spintronics and twistronics^1^, electronic and tunable optical features^2,3^. TMDCs are denoted by the formula $\textrm{MX}_2$, where *M* indicates a transition metal as molybdenum or tungsten $(M = Mo, W)$ which is connected to two *X* atoms corresponding to the chalcogens such as *S*, *Se*,  or *Te*. A bulk TMDC is a layerd material with the weak Van der Waals forces as the dominant force between the layers, which permits to obtain a thin film or a single layer of it by the exfoliation. The number of layers and the arrangement of atoms in adjacent layers in a TMDC specimen determines its electronic and optical properties. TMDCs possess a variety of polytypic structures namely 2*H*, 1*T*, $1T'$, and $T_d$, which are different in the arrangement of atoms. The 2*H* phase with triangular lattice is a direct (indirect) band gap semiconductor in monolayer (bulk) form. Whereas, in the 1*T* phase the chalcogenide atoms are arranged as a hexagon around the metal atom. Due to the instability of 1*T* phase in free-standing form, the structure tends to undergo a spontaneous lattice distortion through the dimerization of transition metal atoms along one of the lattice directions, which results in anisotropic electronic properties^4^. The $1T'$ and $T_d$ phases are structurally similar to the distorted 1*T* phase, and the difference in mirror structure between them can only be recognized in the multilayer films. $\textrm{WTe}_2$ monolayer in $1T'$ phase is the only one among the TMDCs that establishes quantum spin Hall insulator phase, which has been proved with adequate experimental evidences via measurement of quantized edge conductance^5^, and the edge states^6,7^. In particular, $T_d$ phase with the broken inversion symmetry has led to peculiar phenomena. For instance, the multilayer of $\textrm{T}_{\textrm{d}}$–$\textrm{WTe}_2$ has been revealed as a type-II topological Weyl semimetal with tilted Weyl cones^8^, representing a pressure-induced superconductivity^9^, abnormal and giant magnetoresistance effects^10^, extremely high mobility^11^, and low-energy optical absorption^12^.

An in-plane hyperbolic material possesses a highly anisotropic permittivity tensor, such that the real parts of two in-plane principal components of its permittivity tensor have opposite signs^13^. It means that in one direction they behave like a dielectric with a positive permittivity, while in the other direction, they reveal metallic features with negative permittivity. These materials become more noteworthy by considering their tunability via chemical doping, gating, and strain^14^ or temperature^15^. It was predicted that some of the anisotropic 2D materials present hyperbolic surface plasmon polaritons but have not been confirmed experimentally yet^16^. Recently, $\textrm{T}_{\textrm{d}}$–$\textrm{WTe}_2$ thin film has been reported as a semimetal with in-plane anisotropy due to the interplay of its intraband and interband electronic transitions^8,17–19^. In other words, both free carrier response and bound interband transitions characterize the amount of anisotropy that leads to intrinsic tunability. Indeed, hyperbolic plasmons have been realized in exfoliated $\mathrm {WTe_2}$ thin films in a definite frequency range (429–632 $\hbox {cm}^{-1}$). Moreover, the hyperbolic properties can be modified by the temperature^20^. It has been demonstrated that increasing temperature alters the hyperbolic regime of $\textrm{T}_{\textrm{d}}$–$\textrm{WTe}_2$ and its in-plane anisotropy of the optical response^14^. It suggests that $\textrm{T}_{\textrm{d}}$–$\textrm{WTe}_2$ is a perfect and promising hyperbolic material for planar optoelectronics and nanophotonics applications.

Manipulating the polarization state of an incident electromagnetic wave plays a fundamental role in many communication systems, including antennae, satellites, optical fiber, etc. For this aim, many structures based on artificial hyperbolic metamaterials developed with intricate fabrication methods that rely on periodic sub-wavelength features patterned using laborious lithography techniques^21–26^. From a practical perspective, controlling the polarization state of electromagnetic waves by twisting the layers leads to eliminating the symmetry of the structure, and some designs based on twisted metasurfaces have been utilized accordingly^27,28^. On the other hand, the natural in-plane hyperbolicity offers new possibilities for birefringent optical components and polarization-dependent photonics. Compared to the metamaterials, $\textrm{T}_{\textrm{d}}$–$\textrm{WTe}_2$ possesses some characteristics, including easy fabrication, miniaturized structure, and in-plane anisotropy, which makes it distinctive for the manufacturing polarization converters and rotators. To the best of our knowledge, manipulating the light propagation and its polarization state utilizing the twisted TMDC thin films has not been explored. Motivated by this and considering that $\textrm{WTe}_2$ can be assembled into a layered heterostructure, we propose a three-layer structure composed of twisted $\textrm{WTe}_2$ thin films for achieving anisotropic reflection and transmission spectrum. The proposed structure qualifies to change the tilt angle of the linear or elliptic polarization of transmitted or reflected waves through twisted TMDC thin films. In addition to converting linear polarization to an elliptical one, this structure can rotate the linear polarization of the incident wave to any desired direction. The ability to manipulate the polarization state of the incident wave offered by this design, provides a lithography free design with highly efficient and facile mechanism for various applications such as radar detection, communication systems, imaging, and information processing.

The rest of the paper is organized as the following. In Section “Model and Formulas”, the formalisms of the generalized $4\times 4$ transfer-matrix method (TMM) are presented to study $\textrm{WTe}_2$ thin film, and then this method is utilized to study wave propagation in the layered structures. Section “Numerical Results and Discussions” is dedicated to discussing the main results involving the investigating the effect of TMDC layers twisting on the polarization state of the transmitted and reflected waves. Eventually, the main results are summarized in the section of the conclusion.

## Model and Formulas

**Figure 1:**
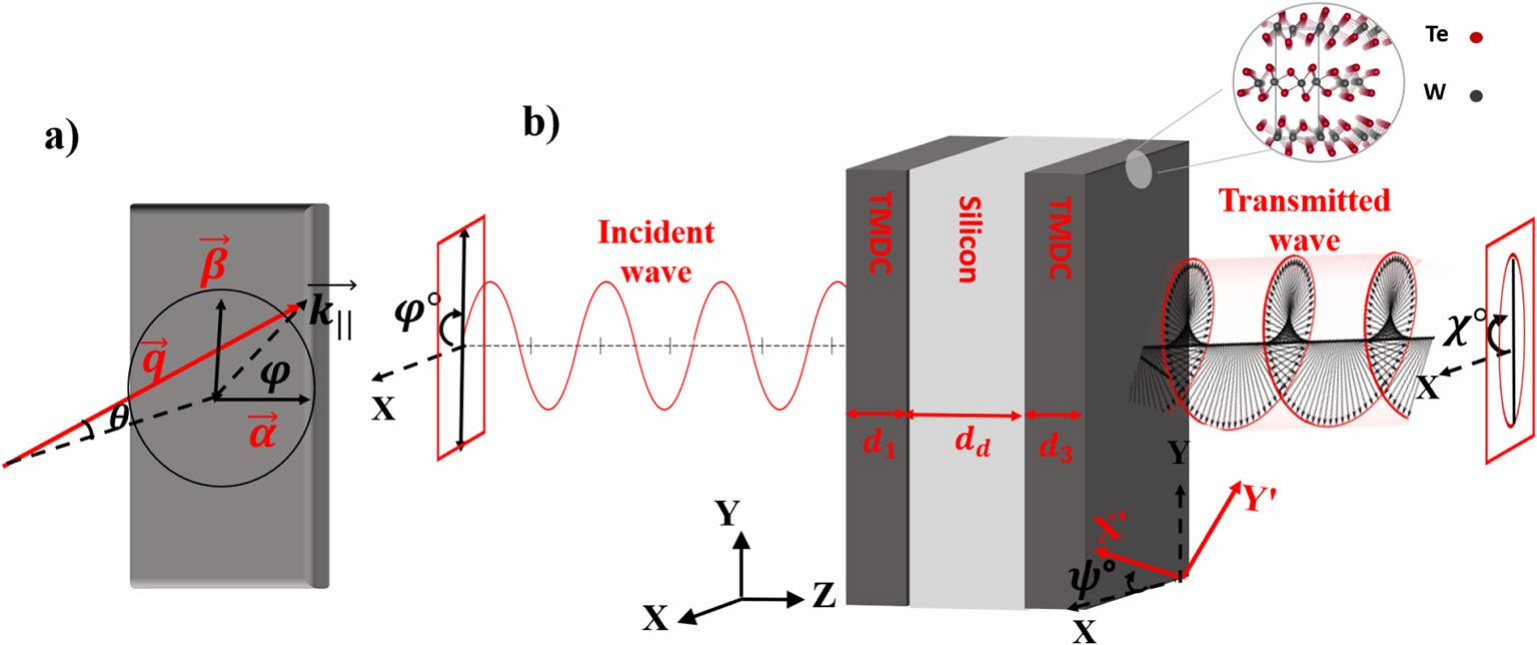
(**a**) Presentation of the components of the incident wave, and (**b**) Schematic representation of the proposed three layer structure composed of two TMDC thin films with in-plain twist angle $\psi$ with respect to each other and separated by a dielectric layer. (**b**) also represents the polarization state of the transmitted wave which is an elliptical polarization.

$\textrm{WTe}_2$ is a layered TMDC whose physical properties depend on the number of layers. The electronic and optical features of monolayers and multilayers of $\textrm{WTe}_2$ have been investigated by *ab-initio* calculations based on density functional theory (DFT)^29^. Here, we only concentrate on the $T_d$ phase of $\textrm{WTe}_2$ due to its inherent hyperbolic characteristic^20^. The tunability of the hyperbolic frequency range is highly desirable to realize various applications including low-loss light modulation at the nanoscale level. The geometry of the proposed system is presented in Fig. 1, which is a three layer structure composed of two thin films of $\textrm{WTe}_2$ with in-plane twist angle of $\psi$ with respect to each other and separated by a dielectric layer with thickness $d_d$. It is assumed that the interfaces between distinct layers are located at the $x-y$ plane and the *z* axis is orthogonal to them. The proposed system is considered in an air background. The tangential component of the incident wave vector can be written as follows, $\textbf{k}_{\Vert }=q \cos (\phi )\hat{i}+q \sin (\phi )\hat{j}=\alpha \hat{i}+\beta \hat{j}$ where $q=\dfrac{\omega }{c}\sin (\theta )$, $\phi$ is the azimuthal angle which determines the angle of the plane of incidence with respect to the *x*-axis. The normal component of the wave vector is given by $k_\bot =\kappa =\dfrac{\omega }{c}\cos (\theta )$, where $\theta$ is the angle of incidence with respect to the *z*-axis.

**Figure 2:**
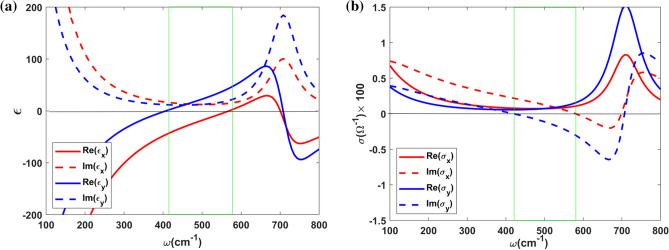
Real and imaginary parts of (**a**) dielectric function and (**b**) optical conductivity along x and y axes of $\textrm{WTe}_2$ thin film with thickness $d=30\hbox { nm}$.

As is well known, a thin film of $\textrm{T}_{\textrm{d}}$–$\textrm{WTe}_2$ has an anisotropic optical response which originates from interfere of two distinct types of the electronic transitions. Off-diagonal terms vanish in its permittivity tensor denoted by $\hat{\varepsilon }_T$, while the diagonal terms are given by^20^:

1 $$\begin{aligned} \begin{array}{l} \varepsilon _{j}=\varepsilon _\infty +\dfrac{i \pi \sigma _{j}}{\varepsilon _0 \omega d},~~~j=x,y\\ \varepsilon _{z}=\varepsilon _\infty , \end{array} \end{aligned}$$

where, $\omega$ is the angular frequency, $\varepsilon _0$ denotes the permittivity of the vacuum, $\varepsilon _\infty$ is the dielectric constant of the environment in the limit of high frequencies, and *d* is the thickness of the thin film. In order to determine the normal permittivity component, $\varepsilon _{z}$, it is assumed that normal electric field cannot excite any current in the thin film of $\textrm{WTe}_2$, thus it is given by the background dielectric constant. The optical conductivity $\sigma$ has contributions from intraband (Drude response) and interband (bound states) transitions denoted respectively by the first and second terms of the following expression^20^,

2 $$\begin{aligned} \sigma _{j}(\omega )=\dfrac{i}{\pi }\dfrac{D_{j}}{\omega +i\Gamma }+\dfrac{i}{\pi }\dfrac{\omega S_{j}}{\omega ^2-\omega _b^2+i\omega \gamma }, \end{aligned}$$

where, *D* and *S* are defined as the spectral weights of intraband and interband transitions, respectively. Furthermore, $\Gamma$ is the intraband transition scattering width, while $\gamma$ indicates the interband transition scattering width and $\omega _b$ denotes the frequency of interband resonance. The hyperbolic frequency range is characterized by the anisotropy of intraband and interband transitions along the principal axes. The parameters in Eq. 2 for an unpatterned thin film of $\textrm{T}_{\textrm{d}}$–$\textrm{WTe}_2$ are given as follows^20^, $D_{x}=8.08\times 10^{11}\,\Omega ^{-1}\hbox { s}^{-1}$, $D_{y}=4.49\times 10^{11}\,\Omega ^{-1}\hbox { s}^{-1}$, $S_{x}=4.31\times 10^{11}\,\Omega ^{-1}\hbox { s}^{-1}$, $S_{y}=8.07\times 10^{11}\,\Omega ^{-1}\hbox { s}^{-1}$, $\Gamma =\gamma =70\hbox { cm}^{-1}$ in 20*K*, $\omega _b=710\hbox { cm}^{-1}$, $\varepsilon _{\infty }=3.3$. Employing these parameters, we have plotted in Fig. 2 the real and imaginary parts of *x* and *y* components of the dielectric function and the optical conductivity of $\textrm{WTe}_2$ thin film with a thickness $d=30\,\hbox {nm}$ in terms of the frequency. As is clear from the figure, in the frequency range denoted by vertical green lines the real part of the *x* component of the dielectric function is negative, while it is positive for *y* component, and it is reversed for the components of the optical conductivity. Therefore, a thin film of $\textrm{WTe}_2$ reveals the dielectric behavior in the *y* direction and metallic properties in the *x* direction in the hyperbolic frequency range. This feature represents the hyperbolic property of the thin film of $\textrm{WTe}_2$. Besides, the real and imaginary parts of *x* and *y* components of the dielectric function of $\textrm{WTe}_2$ thin film have distinct values at all frequencies. Therefore, we expect that a thin film of $\textrm{WTe}_2$ possessing such properties shows fully anisotropic reflection and transmission spectrum together with a high rotation in the polarization direction of the reflected and transmitted waves.

To acquire high efficiency in controlling the amplitude and polarization state of the reflected and transmitted waves, we introduce an exotic structure composed of two TMDC layers with an in-plane twist with respect to each other and connected via a high refractive index dielectric layer with thickness $d_d$. We propose that the second TMDC layer has rotated by an angle $\psi$ around the *z* axis with respect to the first one. We consider a silicon dielectric layer with an almost constant refractive index, $n_{Si}=3.46$, for the frequency range in question. Hence, the dielectric tensor of the second TMDC thin film is obtained by applying a rotation around the *z* axis to the dielectric function of the first TMDC thin film,

3 $$\begin{aligned} \hat{\varepsilon '}_T=R(\psi ) \hat{\varepsilon _T} R^{-1}(\psi )=\left( {\begin{array}{*{20}{c}} {\varepsilon '_x}&{}{\varepsilon _r}&{}{0}\\ {\varepsilon _r}&{}{\varepsilon '_y}&{}{0}\\ {0}&{}{0}&{}{\varepsilon _z} \end{array}} \right) , \end{aligned}$$

where, $R(\psi )$ is the rotation matrix around *z* axis by angle $\psi$, and here we have defined the following parameters,

4 $$\begin{aligned} \begin{array}{l} \varepsilon _{r}={(\varepsilon _y-\varepsilon _x )\sin {\psi }\cos {\psi }}, \\ \varepsilon '_{x}={\varepsilon _x {\cos ^2{\psi }}+\varepsilon _y {\sin ^2{\psi }}}, \\ \varepsilon '_{y}={\varepsilon _x {\sin ^2{\psi }}+\varepsilon _y {\cos ^2{\psi }}}. \end{array} \end{aligned}$$

In order to calculate the reflectance and transmittance of this anisotropic structure the well-known generalized transfer matrix method (TMM) is utilized^30^. The vector of the electric field is proposed in the following form,

5 $$\begin{aligned} \begin{array}{l} \textbf{E}=\textbf{E}_0\exp ^{i[\alpha x+\beta y +\kappa z-\omega t]}. \end{array} \end{aligned}$$

A set of three linear equations for components of the electric field is obtained by writing the wave equation in the twisted TMDC layer which in the matrix form reads,

6 $$\begin{aligned} \left( {\begin{array}{*{20}{c}} {-\beta ^2-\kappa ^2+{k_{0}}^2\varepsilon '_{x}}&{}{\alpha \beta +{k_{0}}^2\varepsilon _{r}}&{}{\alpha \kappa },\\ {\alpha \beta +{k_{0}}^2\varepsilon _{r}}&{}{-\alpha ^2-\kappa ^2+{k_{0}}^2\varepsilon '_{y}}&{}{\beta \kappa },\\ {\alpha \kappa }&{}{\beta \kappa }&{}{-\alpha ^2-\beta ^2+{k_{0}}^2\varepsilon _{z}} \end{array}} \right) \left( {\begin{array}{*{20}{c}} {E_x}\\ {E_y}\\ {E_z} \end{array}} \right) =0, \end{aligned}$$

where, $k_0=\omega /c$ is the wave vector in the vacuum. Setting the determinant of the matrix of the coefficients equal to zero, we find four distinct solutions for the *z* component of the wave vector. Consequently, we have two solutions indicated by $\kappa _1$ and $\kappa _2$ for forward propagation and two for backward one given by $\kappa _3=-\kappa _1$ and $\kappa _4=-\kappa _2$. These solutions are given by the following relations,

7 $$\begin{aligned} \kappa ^{2}_{1,2} =-\dfrac{1}{2\varepsilon _{z}}\left( \Delta \pm \sqrt{\Delta ^2-4 \varepsilon _{z} \left( \alpha ^2 \varepsilon '_{x}+\beta ^2\varepsilon '_{y}-k_0^2\varepsilon '_{x}\varepsilon '_{y}+ 2\alpha \beta \varepsilon _{r}+k_0^2\varepsilon _{r}^2\right) \left( \alpha ^2+\beta ^2-k_0^2\varepsilon _{z}\right) }\right) , \end{aligned}$$

here we have defined,

8 $$\begin{aligned} \Delta =\beta ^{2} ({\varepsilon '_{y}+\varepsilon _{z}})+\alpha ^{2}({\varepsilon '_{x}+\varepsilon _{z}})- k_0^2\varepsilon _{z}({\varepsilon '_{x}+\varepsilon '_{y}})+2\alpha \beta \varepsilon _{r}. \end{aligned}$$

It is obvious that by setting $\psi =0$ we will obtain the solutions of the *z* components of the wave vector in the fixed TMDC thin film. For dielectric layer, it is enough to set all of three components of the dielectric tensor equal to the dielectric constant of the medium.

The general solution for the wave equation inside the thin film of the anisotropic $\textrm{WTe}_2$ must be in the form of a linear combination of the four eigenmodes of the wave equation given by Eq. 7. Hence, we can write,

9 $$\begin{aligned} \begin{array}{l} \textbf{E}(\textbf{r},\omega )=\sum \limits _{j=1}^4\varvec{\Gamma }_{\textbf{j} }E_j\exp ^{i[\alpha x+\beta y +\kappa _{j}z-\omega t]}, \end{array} \end{aligned}$$

where, $\varvec{\Gamma }_{\textbf{j} }$ are eigenvectors corresponding to the four $\kappa _{j}$ solutions obtained from the wave equation, Eq. (6), and $E_j$ are coefficients of distinct solutions. Then, by applying the boundary conditions on the tangential components of the electric and magnetic fields, the following matrix relation is obtained between coefficients of the electric filed in adjacent layers,

10 $$\begin{aligned} T_{n-1}\left[ {\begin{array}{*{20}{c}} {E^{t}_x}\\ {E^{t}_y}\\ {E^{r}_x}\\ {E^{r}_y} \end{array}} \right] _{n-1}=T_nP_n \left[ {\begin{array}{*{20}{c}} {E^{t}_x}\\ {E^{t}_y}\\ {E^{r}_x}\\ {E^{r}_y} \end{array}} \right] _{n}, \end{aligned}$$

where, superscripts *t* and *r* denote amplitudes of the forward and backward waves, respectively. The dynamical and propagation matrices in the above equation are defined as,

11 $$\begin{aligned} \begin{array}{l} T_{n}=\left[ {\begin{array}{*{20}{c}} {\Gamma _{1x}}&{}{\Gamma _{2x}}&{}{\Gamma _{3x}}&{}{\Gamma _{4x}} \\ {\Gamma _{1y}}&{}{\Gamma _{2y}}&{}{\Gamma _{3y}}&{}{\Gamma _{4y}} \\ {\kappa _{1n}\Gamma _{1x}-\alpha \Gamma _{1z}}&{}{\kappa _{2n}\Gamma _{2x}-\alpha \Gamma _{2z}} &{}{\kappa _{3n}\Gamma _{3x}-\alpha \Gamma _{3z}}&{}{\kappa _{4n}\Gamma _{4x}-\alpha \Gamma _{4z}} \\ {\beta \Gamma _{1z}-\kappa _{1n}\Gamma _{1y}}&{}{\beta \Gamma _{2z}-\kappa _{2n}\Gamma _{2y}}&{} {\beta \Gamma _{3z}-\kappa _{3n}\Gamma _{3y}}&{}{\beta \Gamma _{4z}-\kappa _{4n}\Gamma _{4y}}\\ \end{array}} \right] ,\\ \\ P_{n}=\left[ {\begin{array}{*{20}{c}} {\exp [{-i\kappa _{1n}d_n}]}&{}{0}&{}{0}&{}{0}\\ {0}&{}{\exp [{-i\kappa _{2n}d_n}]}&{}{0}&{}{0}\\ {0}&{}{0}&{}{\exp [{-i\kappa _{3n}d_n}]}&{}{0}\\ {0}&{}{0}&{}{0}&{}{\exp [{-i\kappa _{4n}d_n}]}\\ \end{array}} \right] , \end{array} \end{aligned}$$

where, $d_n$ denotes the width of the *n*th layer. Here, the matrix elements of *T* as they are obtained from the wave equation are given by,

12 $$\begin{aligned} \begin{array}{l} \Gamma _{1x}=-\Gamma _{3x}=\Gamma _{2y}=\Gamma _{4y}=1,\\ \\ \Gamma _{ix}=\dfrac{\alpha \beta \kappa _{i}^2-(\alpha \beta +\varepsilon _r)(-\alpha ^2-\beta ^2+k_0^2\varepsilon _{z})}{(-\alpha ^2-\beta ^2+k_0^2\varepsilon _{z}) (-\beta ^2-{\kappa _{i}}^2+k_0^2\varepsilon '_{x})-\alpha ^{2}{\kappa _{i}}^{2})}\Gamma _{iy},~~~i=2,4~,\\ \\ \Gamma _{iy}=\dfrac{\alpha \beta \kappa _{i}^2-(\alpha \beta +\varepsilon _r)(-\alpha ^2-\beta ^2+k_0^2\varepsilon _{z})}{(-\alpha ^2-\beta ^2+k_0^2\varepsilon _{z}) (-\alpha ^2-{\kappa _{i}}^2+k_0^2\varepsilon '_{y})-\beta ^{2}\kappa _{i}^{2})}\Gamma _{ix},~~~i=1,3~,\\ \\ \Gamma _{iz}=-\dfrac{\alpha \kappa _{i}}{(-\alpha ^2-\beta ^2+k_0^2\varepsilon _{z})}\Gamma _{ix}- \dfrac{\beta \kappa _{i}}{(-\alpha ^2-\beta ^2+k_0^2\varepsilon _{z})}\Gamma _{iy},~~~i=1,2,3,4~.\\ \end{array} \end{aligned}$$

In an isotropic and homogeneous layer, where $\varepsilon _x=\varepsilon _y=\varepsilon _z=\varepsilon$ and hence $\kappa _1=\kappa _2=\kappa$, $\kappa _3=\kappa _4=-\kappa$, the eigenvectors $\Gamma$ given by above equations reduce to $\Gamma _{1y}=\Gamma _{3y}=\Gamma _{2x}=\Gamma _{4x}=0$. Consequently, the total transfer matrix of the structures composed of single and double $\textrm{WTe}_2$ thin film on the *Si* substrate can be written as,

13 $$\begin{aligned} \begin{array}{l} T_{S}=T_{a}^{-1} {T_1}P_1{T_1}^{-1} T_a,\\ T_{D}=T_{a}^{-1} {T_1}P_1{T_1}^{-1}{T_d}P_d{T_d}^{-1}{T_2}P_2{T_2}^{-1} T_a, \end{array} \end{aligned}$$

where, $T_a$ denotes the dynamical matrix for air medium. To find the reflectance and transmittance through the structure we apply the following transformation between the incident and outgoing components of the electric field^30,31^,

14 $$\begin{aligned} \left[ {\begin{array}{*{20}{c}} {E^{i}_{x}}\\ {E^{r}_{x}}\\ {E^{i}_{y}}\\ {E^{r}_{y}} \end{array}} \right] ={\Lambda }^{-1} T {\Lambda } \left[ {\begin{array}{*{20}{c}} {E^{t}_{x}}\\ {0}\\ {E^{t}_{y}}\\ {0} \end{array}} \right] = T^* \left[ {\begin{array}{*{20}{c}} {E^{t}_{x}}\\ {0}\\ {E^{t}_{y}}\\ {0} \end{array}} \right] , \end{aligned}$$

where, $E^{i}_{x,y}$ denote the incident electric field components and $\Lambda$ is given by,

$$\Lambda =\left( \begin{matrix} 1&{}0&{}0&{}0\\ 0&{}0&{}1&{}0\\ 0&{}1&{}0&{}0\\ 0&{}0&{}0&{}1\end{matrix}\right) .$$

The reflection and transmission coefficients for an incident wave with electric field in the *x* direction are given as follows^32^,

15 $$\begin{aligned} \begin{array}{l} r_{xx}=\dfrac{E^{r}_{x}}{E^{i}_{x}}\vert _{E^{i}_{y}=0}= \dfrac{T^*_{21}T^*_{33}-T^*_{23}T^*_{31}}{T^*_{11}T^*_{33}-T^*_{13}T^*_{31}},~~~ t_{xx}=\dfrac{E^{t}_{x}}{E^{i}_{x}}\vert _{E^{i}_{y}=0}= \dfrac{T^*_{33}}{T^*_{11}T^*_{33}-T^*_{13}T^*_{31}}, \\ r_{xy}=\dfrac{E^{r}_{y}}{E^{i}_{x}}\vert _{E^{i}_{y}=0}= \dfrac{T^*_{41}T^*_{33}-T^*_{43}T^*_{31}}{T^*_{11}T^*_{33}-T^*_{13}T^*_{31}},~~~ t_{xy}=\dfrac{E^{t}_{y}}{E^{i}_{x}}\vert _{E^{i}_{y}=0}= \dfrac{-T^*_{31}}{T^*_{11}T^*_{33}-T^*_{13}T^*_{31}}. \end{array} \end{aligned}$$

Whereas, for incident wave with electric field in the *y* direction these coefficients are given by,

16 $$\begin{aligned} \begin{array}{l} r_{yy}=\dfrac{E^{r}_{y}}{E^{i}_{y}}\vert _{E^{i}_{x}=0}= \dfrac{T^*_{11}T^*_{43}-T^*_{41}T^*_{13}}{T^*_{11}T^*_{33}-T^*_{13}T^*_{31}},~~~ t_{yy}=\dfrac{E^{t}_{y}}{E^{i}_{y}}\vert _{E^{i}_{x}=0}= \dfrac{-T^*_{11}}{T^*_{11}T^*_{33}-T^*_{13}T^*_{31}}, \\ r_{yx}=\dfrac{E^{r}_{x}}{E^{i}_{y}}\vert _{E^{i}_{x}=0}= \dfrac{T^*_{11}T^*_{23}-T^*_{21}T^*_{13}}{T^*_{11}T^*_{33}-T^*_{13}T^*_{31}},~~~ t_{yx}=\dfrac{E^{t}_{x}}{E^{i}_{y}}\vert _{E^{i}_{x}=0}= \dfrac{T^*_{13}}{T^*_{11}T^*_{33}-T^*_{13}T^*_{31}}. \end{array} \end{aligned}$$

Eventually, the components of the reflectance and transmittance can be obtained as $R_{i,j}=\vert r_{ij}\vert ^2$, $T_{i,j}=\vert t_{ij}\vert ^2$, with $i,j=x,y$. Moreover, we define the total reflectance and transmittance in the *x* and *y* directions by $R_{j}=\vert r_{jj}\vert ^2+\vert r_{ji}\vert ^2$ and $T_{j}=\vert t_{jj}\vert ^2+\vert t_{ji}\vert ^2$. In the following section, we present the results obtained by this model and theoretical approach.

## Numerical Results and Discussions

### Optical responses of single and double $\textrm{WTe}_2$ thin film

**Figure 3:**
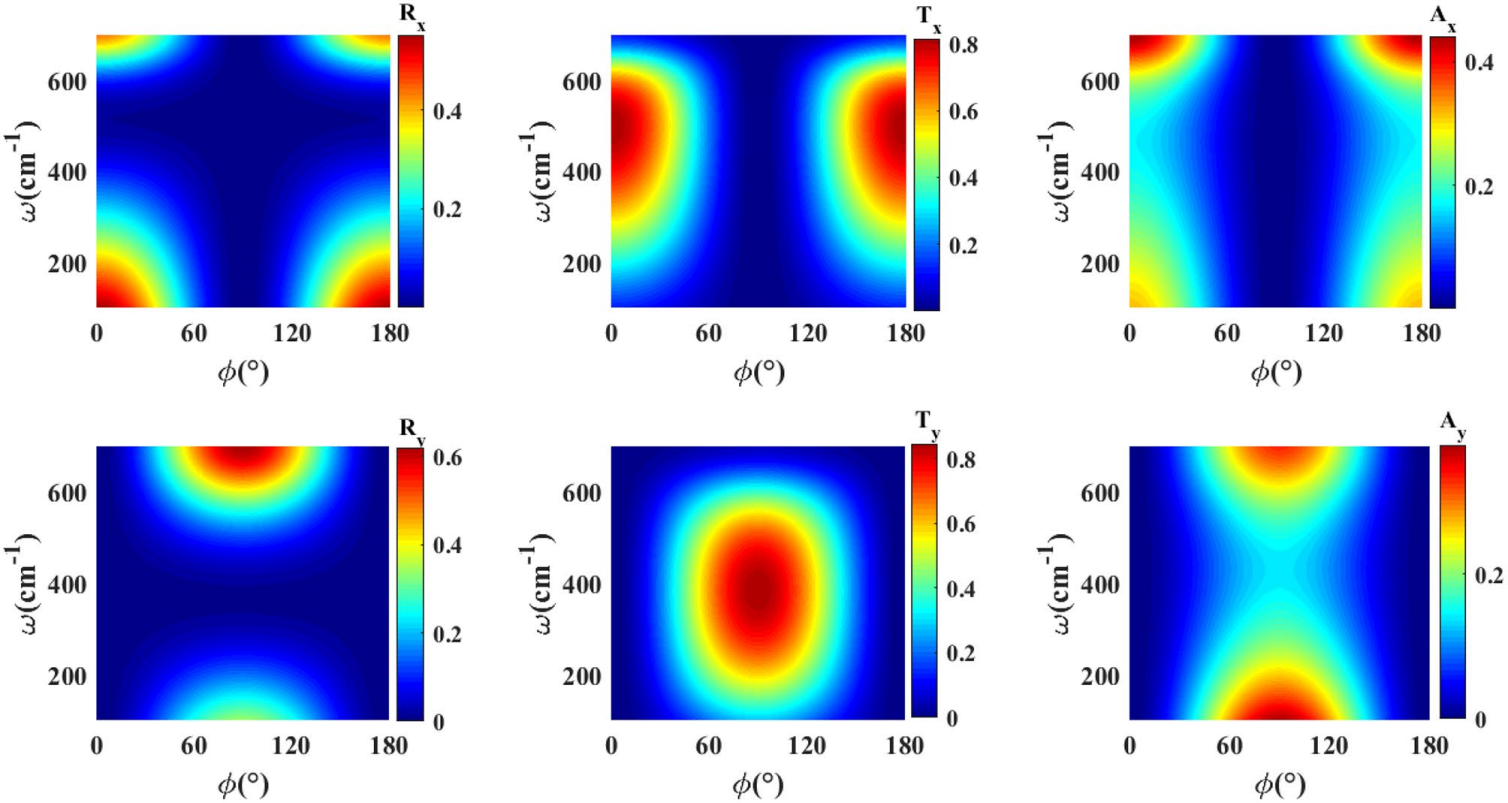
Colormaps of reflectance, transmittance and absorption for *x* and *y* components of the electric field incident on the single $\textrm{WTe}_2$ thin film as a function of frequency $\omega$ and polarization angle $\phi$. The thicknesses of $\textrm{WTe}_2$ thin film and *Si* layer are $d=30\hbox { nm}$, $d_d=100\hbox { nm}$, respectively.

Let us first study wave propagation through a single $\textrm{WTe}_2$ thin film deposited on a Si substrate. The thicknesses of the $\textrm{WTe}_2$ thin film and Si layer are considered to be 30 nm and 100 nm, respectively. Since we expect that the essential features of the considered structures are revealed in the normal incidence configuration, so to prevent unnecessary complexity, we set $\theta =0$ in all of the subsequent calculations. The reflectance, transmittance, and absorption for the *x* and *y* components of the electric field of a wave incident normally on a single $\textrm{WTe}_2$ thin film as a function of frequency and angle of the electric field with respect to the *x* axis is shown in Fig. 3. As is expected, the optical response depends on the angle of the incident polarization with respect to the optical axis *x* of $\textrm{WTe}_2$ thin film. However, the reflectance and transmittance spectrums of the wave components show approximately opposite dependence on $\phi$, as is expected to happen by changing the polarization direction of the incident wave. This dependence is more profound for the frequency range of approximately 400–$600\hbox { cm}^{-1}$ where the hyperbolic behavior reveals itself. We see a deep in reflectance in this frequency range, whereas a peak is observed in the transmittance (see the supplementary information). As is clear from Fig. 3, the *x* polarized ($\phi =0^\circ , 180^\circ$) and *y* polarized ($\phi =90^\circ$) waves represent completely different behavior in the whole range of frequency due to the distinct permittivity components of $\textrm{WTe}_2$ thin film in these directions. For clearness, we have examined the frequency and polarization angle dependence of the reflectance and transmittance of the bare silicon substrate and did not find such frequency and polarization angle dependence (see for details the supplementary information).

Moreover, an incident wave with *x* or *y* polarization does not generate reflected and transmitted waves with transverse polarization. While an incident wave with other polarizations results in different components of the transmitted or reflected electric field due to the anisotropic optical response of $\textrm{WTe}_2$ thin film. Furthermore, $\textrm{WTe}_2$ thin film shows high absorbance around the frequencies $100\hbox { cm}^{-1}$ and $700\hbox { cm}^{-1}$, which is consistent with the behavior of the *x* and *y* components of the dielectric tensor presented in Fig. 2. The relatively high amount of the imaginary parts of *x* and *y* components of the dielectric tensor around these frequencies is apparent in this figure.

**Figure 4:**
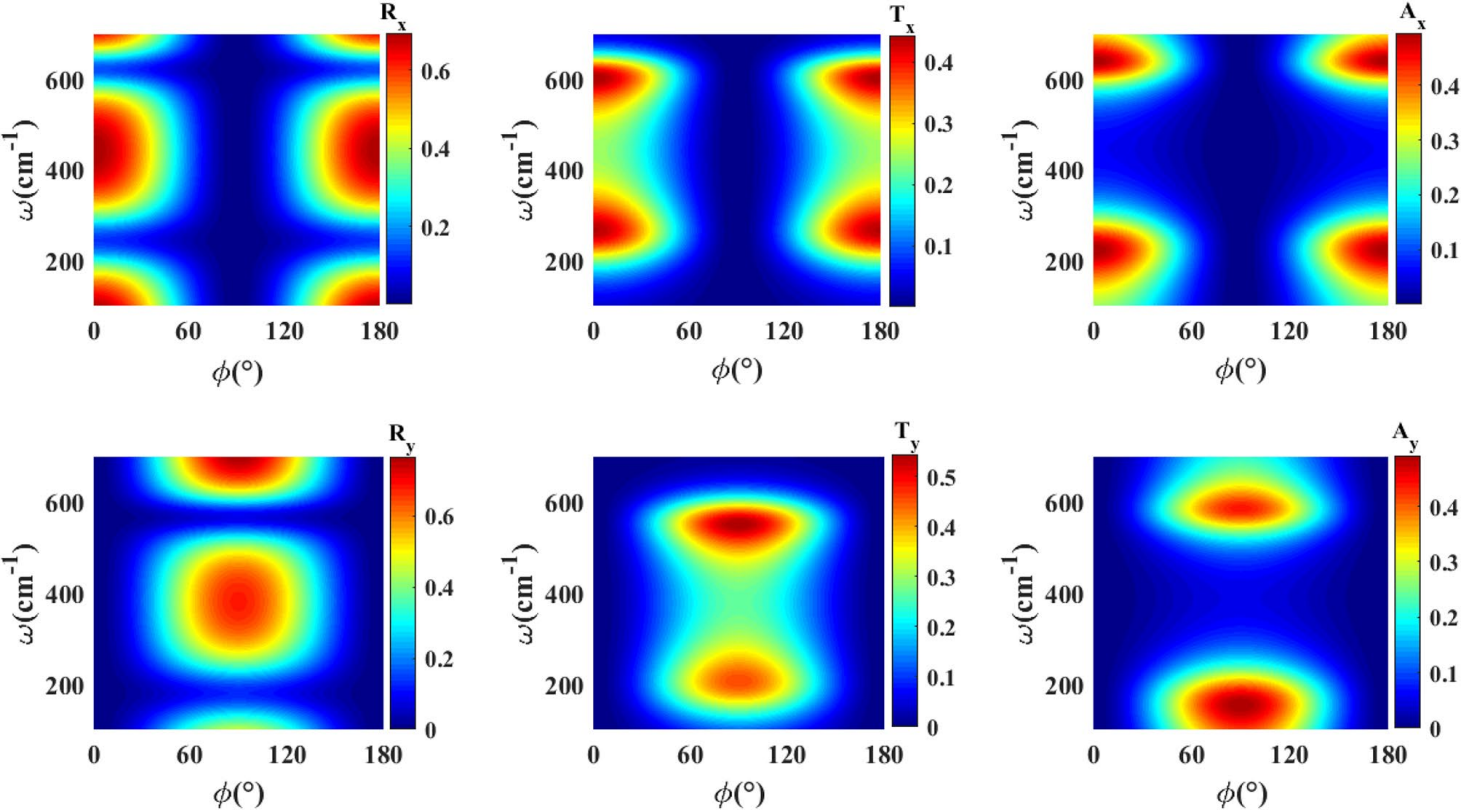
Plots of reflectance, transmittance and absorbance for *x* and *y* components of the electric field incident on the untwisted double $\textrm{WTe}_2$ thin film structure as a function of frequency $\omega$ and polarization angle $\phi$. The thicknesses of $\textrm{WTe}_2$ thin films and *Si* substrate are $d_1=d_2=30\hbox { nm}$, $d_d=2\,\upmu \hbox {m}$, respectively. Here, $\textrm{WTe}_2$ thin films are not twisted with respect to each other, $\psi =0$.

Now we proceed to present the optical response of the double $\textrm{WTe}_2$ thin film structure. We have presented in Fig. 4 the reflectance, transmittance, and absorbance of the double $\textrm{WTe}_2$ thin film structure untwisted with respect to each other ($\psi =0$). Here, thicknesses of both thin films are equal to $d_1=d_2=30\hbox { nm}$ and thickness of the dielectric substrate layer is $d_d=2\,\upmu \hbox {m}$. All of the features indicated for a single thin film structure are presented identically in the double thin film structure. The only difference is the separation of the deep in the reflectance and peak in the transmittance and absorption into two parts. This feature originates from the interferences in the substrate layer.

### Polarization rotation

In this section, we investigate the essential feature of the proposed structures. Generally, the inherent anisotropy of $\textrm{WTe}_2$ thin film gives rise to the conversion of the linearly polarized incident wave to the elliptically polarized reflected and transmitted waves. Indeed, the difference in the principal components of the permittivity of this material results in different optical responses in the *x* and *y* directions as already illustrated. The polarization state of the output waves will certainly depend on the polarization angle and the frequency of the incident wave. For the normal incidence, the azimuthal and ellipticity angles of the polarization ellipse with respect to the *x*-axis for the transmitted and reflected waves are expressed as follows^33^,

17 $$\begin{aligned} \begin{array}{l} \chi =\dfrac{1}{2}\tan ^{-1}\left( \dfrac{2\vert E^{t(r)}_{x}\vert \vert E^{t(r)}_{y}\vert \cos (\delta )}{\vert E^{t(r)}_{x}\vert ^2-\vert E^{t(r)}_{y}\vert ^2} \right) ,\\ \\ {\eta =\dfrac{1}{2}\sin ^{-1}\left( \dfrac{2\vert E^{t(r)}_{x}\vert \vert E^{t(r)}_{y}\vert \sin (\delta )}{\vert E^{t(r)}_{x}\vert ^2+\vert E^{t(r)}_{y}\vert ^2} \right) ,} \end{array} \end{aligned}$$

where, $\delta =\delta _y-\delta _x$ expresses the phase difference between *x* and *y* components of transmitted and reflected waves $E^{t}_{x,y}$ and $E^{r}_{x,y}$ given by,

18 $$\begin{aligned} \begin{array}{l} E^{t}_{x}=\dfrac{-T^*_{33}\cos (\phi )+T^*_{13}\sin (\phi )}{T^*_{31}T^*_{13}-T^*_{11}T^*_{33}}, \\ E^{t}_{y}=\dfrac{T^*_{31}\cos (\phi )-T^*_{11}\sin (\phi )}{T^*_{31}T^*_{13}-T^*_{11}T^*_{33}}, \\ E^{r}_{x}=T^*_{21}E^{t}_{x}+T^*_{23}E^{t}_{y}, \\ E^{r}_{y}=T^*_{41}E^{t}_{x}+T^*_{43}E^{t}_{y}. \end{array} \end{aligned}$$

**Figure 5:**
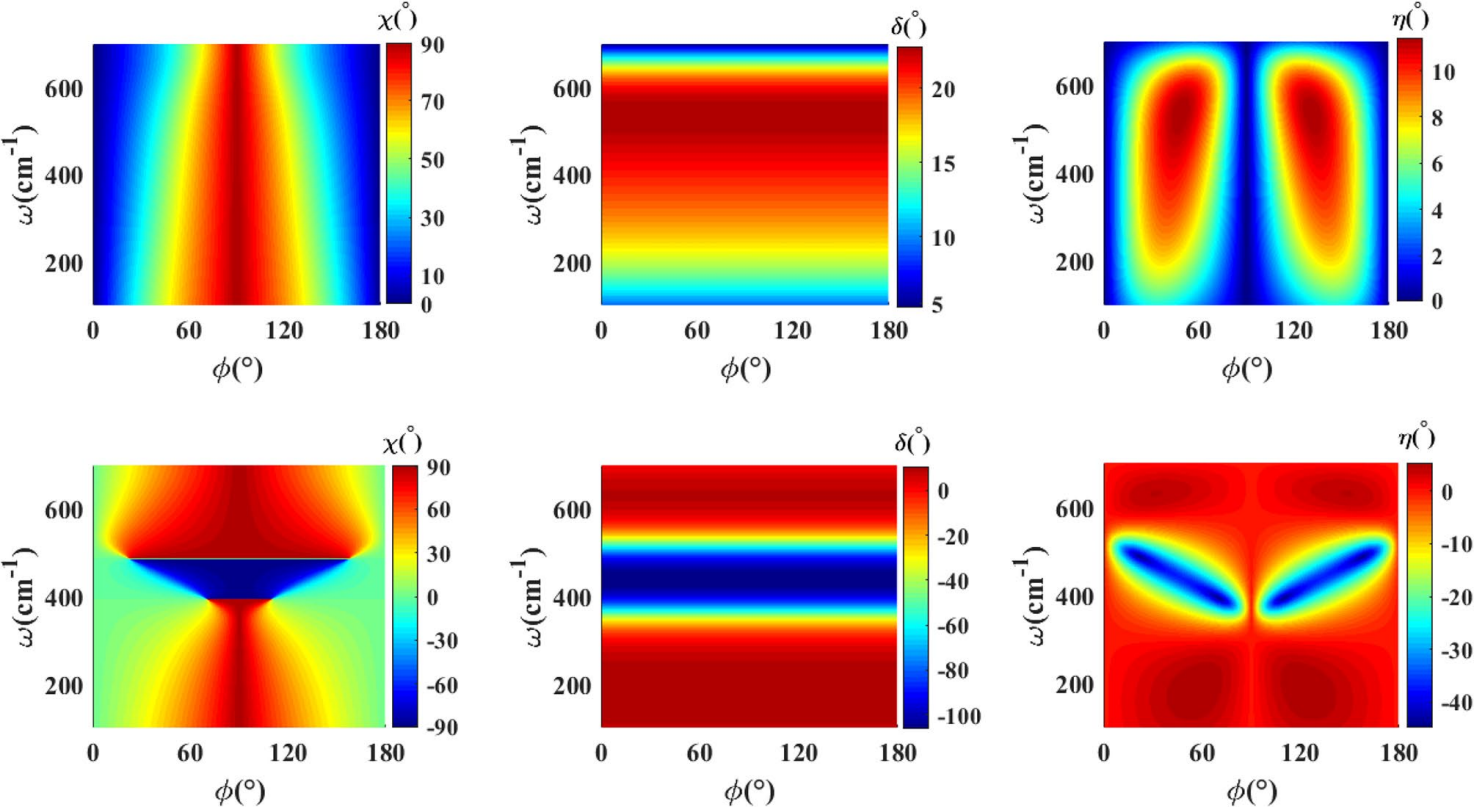
The azimuthal angle of the polarization ellipse ($\chi$), phase difference ($\delta$) and the ellipticity angle ($\eta$) for transmitted (up row) and reflected (down row) waves through a $\textrm{WTe}_2$ thin film with $d=30\hbox { nm}$ as a function of the polarization angle of the incident wave and frequency.

Figure 5 displays the azimuthal angle of the polarization ellipse ($\chi$), the phase difference ($\delta$) between *x* and *y* components of the electric field, and the ellipticity angle ($\eta$) for transmitted (up row) and reflected (down row) waves. Here we have considered the same values for the parameters as those in Fig. 2. It demonstrates that a linearly incident wave, with approximately any frequency, is generally converted to a wave with elliptical polarization. For the transmitted wave, we observe that the azimuthal angle of the polarization ellipse increases by increasing the angle of the incident polarization up to $90^{\circ }$, and then by further increase of $\phi$ toward $180^{\circ }$ it decreases from $90^{\circ }$ to zero. For the mid values of $\phi$, the green regions in the plot of $\chi$, a considerable difference is observed between $\phi$ and $\chi$ values. Nevertheless, this behavior does not demonstrate the polarization rotation due to the small values of the phase difference ($\delta$), and as well due to the nonzero values of the ellipticity angle ($\eta$) in these regions. As is clear from the figures, the phase difference shows negligible dependence on the incident polarization angle, whereas it reflects rigorous relevance on the frequency, especially in the hyperbolic frequency range. In the case of the reflected wave, a crucial variation in the phase difference and correspondingly an inverse behaviour in the azimuthal angle of the polarization ellipse happens in the frequency range around 390–$510\hbox { cm}^{-1}$ lying in the hyperbolic frequency range. In particular, for the reflected wave, there are frequency ranges lying in the hyperbolic regime around 390–$410\hbox { cm}^{-1}$ and 490–$510\hbox { cm}^{-1}$ with $90^{\circ }$ phase difference and approximately $-\pi /4$ ellipticity angle, indicating the nearly circular polarization of the reflected wave.

**Figure 6:**
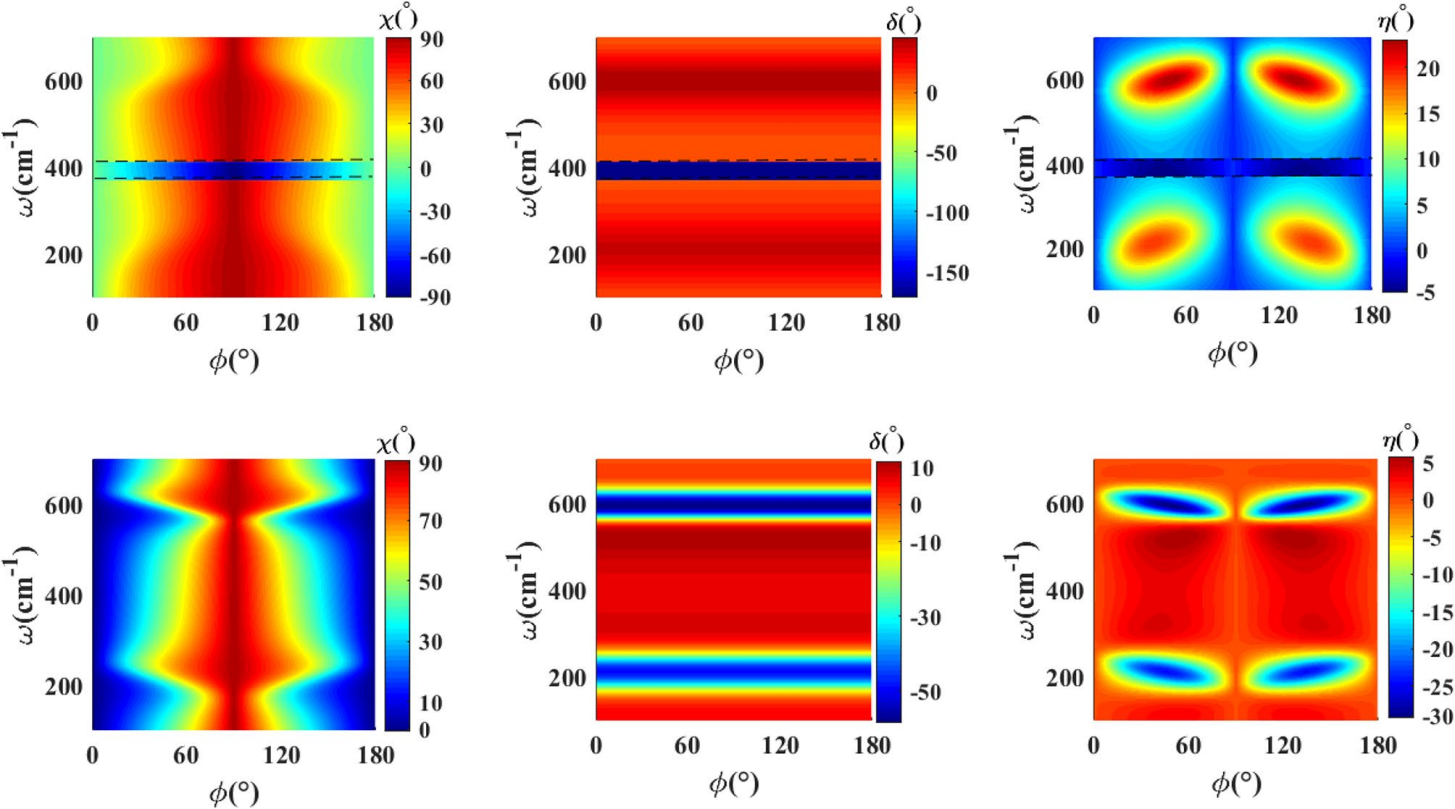
The azimuthal angle of the polarization ellipse ($\chi$), phase difference ($\delta$) and ellipticity angle ($\eta$) as a function of polarization angle of incident wave ($\phi$) and frequency for transmitted (up row) and reflected (down row) for double $\textrm{WTe}_2$ thin film structure with zero twist angle between them ($\psi =0^\circ$), and $d_1=d_2=30\hbox { nm}$, $d_d=2\,\upmu \hbox {m}$.

To acquire a perfect control on the polarization state of the transmitted and reflected waves, we propose a structure composed of double thin film of $\textrm{WTe}_2$ twisted by in-plane angle $\psi$ with respect to each other. Figure 6 represents the polarization state of the transmitted (up row) and reflected (down row) waves through the untwisted double $\textrm{WTe}_2$ thin films structure in terms of frequency and incident polarization angle. We find that as a result of the coupling between two thin films, serious variations happen in the phase difference of the output waves in terms of frequency for every incident polarization angle. In particular, for the transmitted wave, a region of high phase difference with $\delta \sim 171^{\circ }$ appears in the frequency range 380–$410\hbox { cm}^{-1}$, which leads to an inverse behavior of the azimuthal angle of the polarization ellipse and values of the ellipticity angle well close to zero. Therefore, in this range, the polarization state becomes approximately linearly polarized and rotated by $0^{\circ }$–$90^{\circ }$ with respect to the incident wave, depending on the angle of the incident polarization. Hence, this structure generates a polarization rotation approximately in the range $0^{\circ }$–$90^{\circ }$ for the transmitted wave. It is worth mentioning that this effect is generated by a very thin and lithography-free structure.

**Figure 7:**
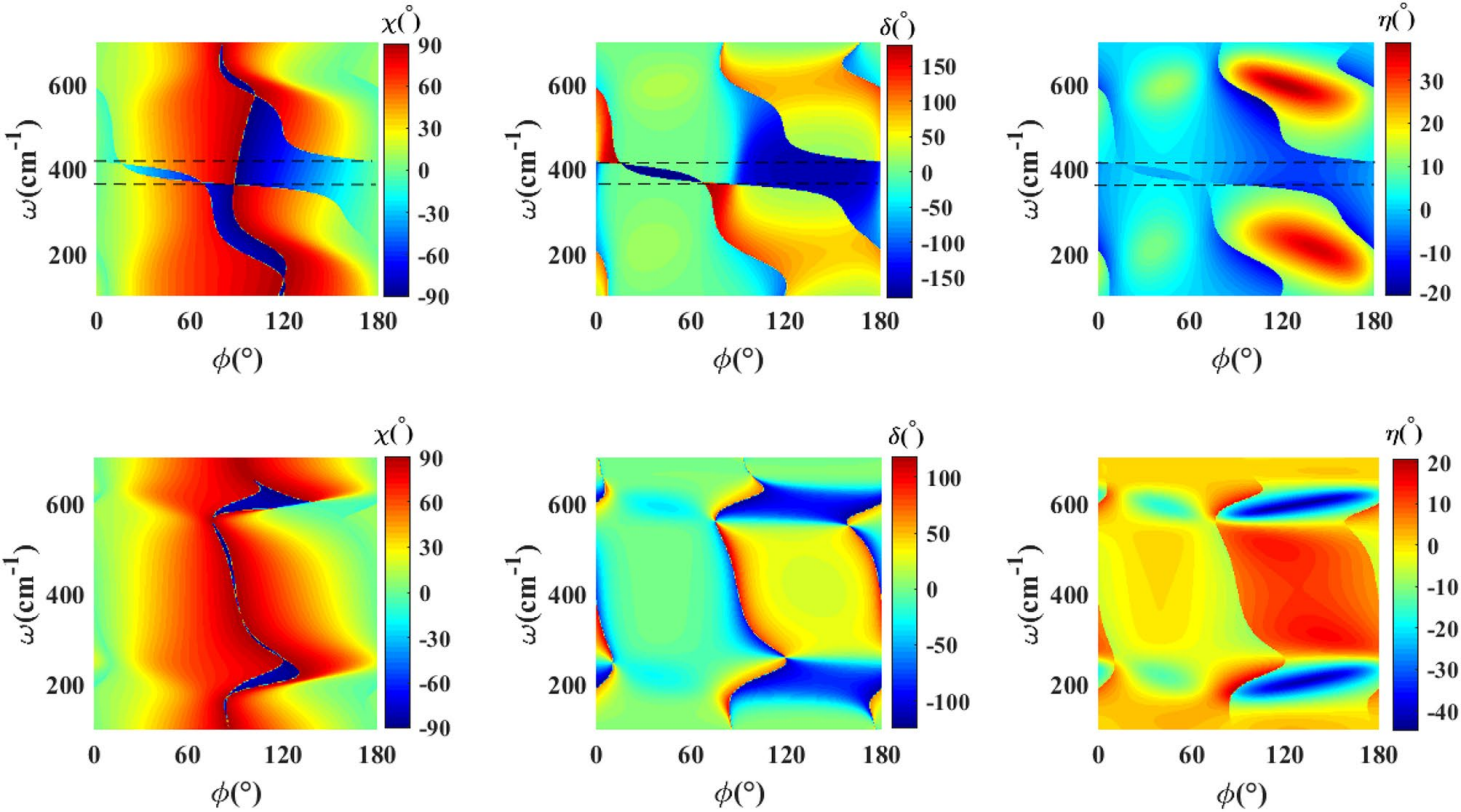
The azimuthal angle of the polarization ellipse ($\chi$), phase difference ($\delta$) and ellipticity angle ($\eta$) as a function of polarization angle of incident wave ($\phi$) and frequency for transmitted (up row) and reflected (down row) for double $\textrm{WTe}_2$ thin film structure with nonzero twist angle between thin films ($\psi =30^\circ$), and $d_1=d_2=30\hbox { nm}$, $d_d=2\,\upmu \hbox {m}$.

Now let us analyze the effect of the twisting two $\textrm{WTe}_2$ thin films with respect to each other. Figure 7 exhibits effect of the twist angle $\psi =30^{\circ }$ on the polarization state of the transmitted (up row) and reflected (down row) waves. As is apparent from the figures, twisting the double $\textrm{WTe}_2$ thin film has an immense effect on the azimuthal angle of the polarization ellipse, phase difference, and ellipticity angle for both transmitted and reflected waves, especially for the incident polarization angles in the range of $90^{\circ }$–$180^{\circ }$. We find a very complex behavior for the polarization state of the output waves in terms of the frequency and incident polarization angle. In the case of the transmitted wave, as is apparent in the figures of up row, there are ranges of frequency and incident polarization angle where this twisted double thin film features approximately $\delta =90^{\circ }$ and $\delta =180^{\circ }$ phase differences. For the incident polarization in the range $20^{\circ }$–$60^{\circ }$ and the frequency range 370–$410\hbox { cm}^{-1}$ a region with high phase difference $\delta \sim 179^{\circ }$ appears, which leads to a high linearly polarized transmitted wave with approximate rotation in the range $40^{\circ }$–$60^{\circ }$ depending on the incident polarization angle. Besides, there is another region with a high phase difference $\delta \sim 170^{\circ }$ for $\phi \simeq 110^{\circ }$–$170^{\circ }$ and $\omega \simeq 370$–$440\hbox { cm}^{-1}$ which can result in the linearly polarized transmitted wave with a rotation in the range $60^{\circ }$–$20^{\circ }$ depending on the incident polarization angle. For the reflected wave, we only find a region of approximately $90^{\circ }$ phase difference and $-\pi /4$ ellipticity angle around the frequencies $\omega =200\hbox { cm}^{-1}$ and $\omega =600\hbox { cm}^{-1}$, which reflects the possibility of the circular polarization of the reflected wave.

**Figure 8:**
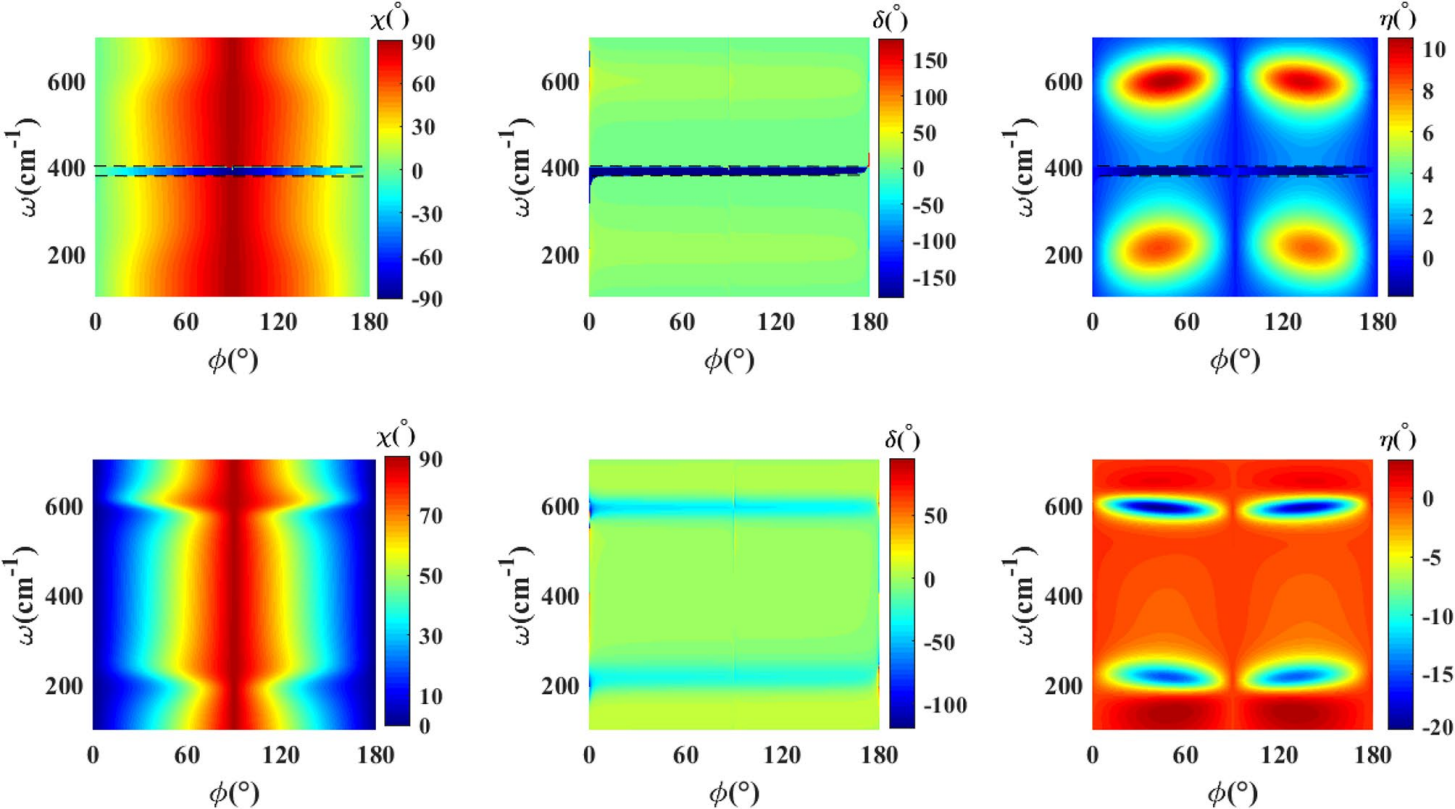
The azimuthal angle of the polarization ellipse ($\chi$), phase difference ($\delta$) and ellipticity angle ($\eta$) as a function of polarization angle of incident wave ($\phi$) and frequency for transmitted (up row) and reflected (down row) for double $\textrm{WTe}_2$ thin film structure with nonzero twist angle between thin films ($\psi =45^\circ$), and $d_1=d_2=30\hbox { nm}$, $d_d=2\,\upmu \hbox {m}$.

Fig. 8 represents the polarization state of the transmitted (up row) and reflected (down row) waves for a twisted double $\textrm{WTe}_2$ thin film structure with twist angle $\psi =45^{\circ }$. The results are similar to the untwisted situation. In this case, we find again a range of frequency $\omega \simeq 380$–$400\,\hbox {cm}^{-1}$ with $\delta \sim 176^{\circ }$ phase difference and inverse behavior of the azimuthal angle of polarization ellipse, which indicates linearly polarized transmitted wave with rotation in the range $0^{\circ }$–$90^{\circ }$. Consequently, we infer that the twisted double thin film structure offers the possibility of tunable polarization conversion and rotation.

Eventually, to complete the investigation of the optical properties, we analyzed the behavior of these structures in terms of the thickness of the silicon substrate and presented them in the supplementary information.

**Figure 9:**
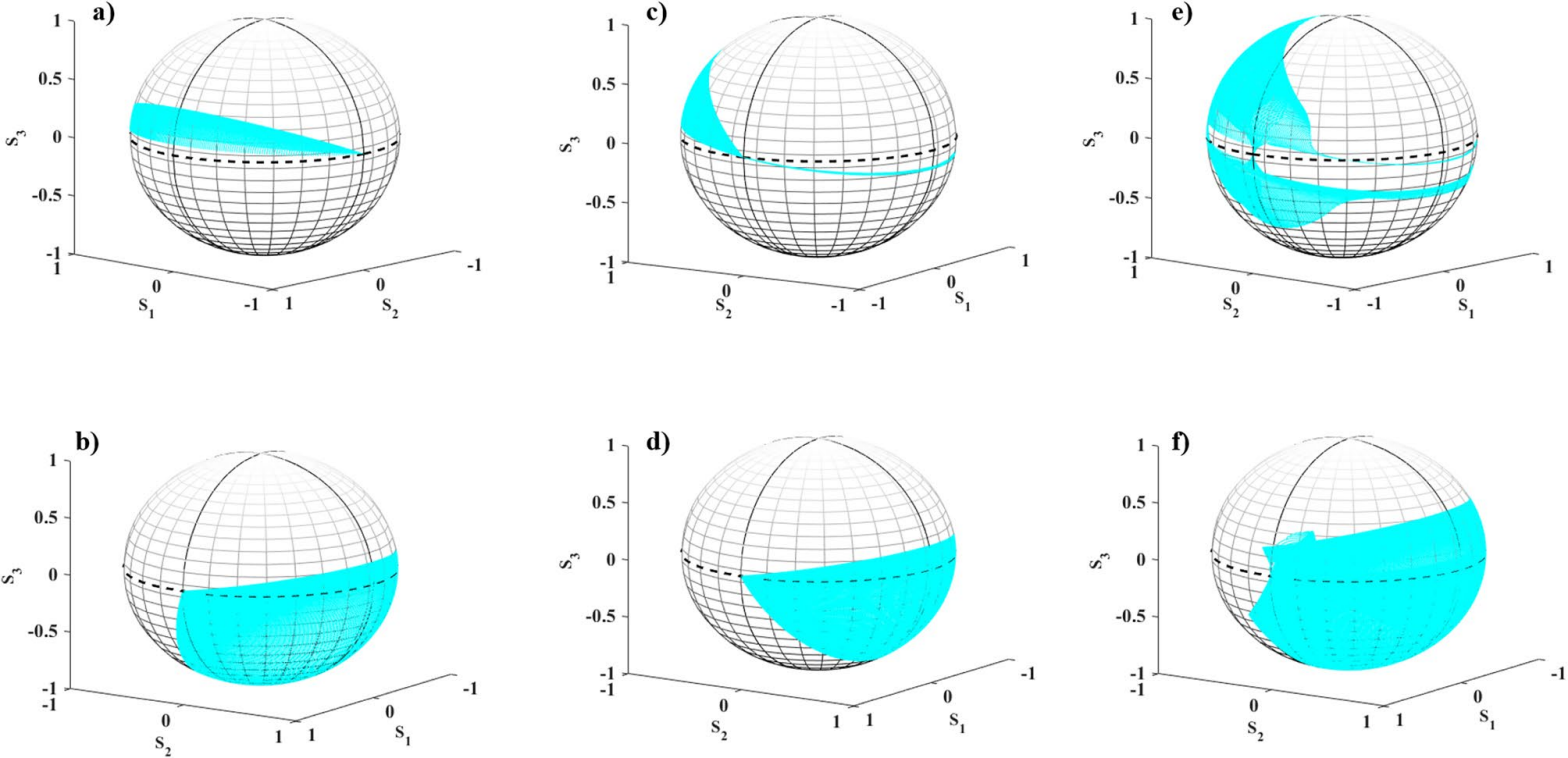
Polarization states on the Poincaré sphere in **(a, b)** for the single $\textrm{WTe}_2$ thin film, **(c, d)** for the double $\textrm{WTe}_2$ thin film structure with $\psi =0^\circ$, and **(e, f)** $\psi =30^\circ$ for transmitted (up row) and reflected (down row) waves.

To the conceivable exhibition of the polarization rotation or conversion in the reflected or transmitted waves, we utilize the intuitive representation of the output polarization states on the Poincaré sphere. It demonstrates the polarization state of the output wave by determining the azimuth and ellipticity angles of the polarization state (see details in the supplementary information). In Fig. 9, the polarization states of transmitted (up row) and reflected (down row) waves in the structures composed of single and double $\textrm{WTe}_2$ thin film are displayed on the Poincaré sphere. As is seen, the polarization states of the single $\textrm{WTe}_2$ thin film indicate only elliptical polarizations for the transmitted wave and represent elliptical polarizations accompanied by the linear (points on the equator) and circular (points on the poles) polarizations for the reflected wave. Whereas, the polarization states of the twisted double $\textrm{WTe}_2$ thin film exhibit points on the Poincaré sphere indicating nearly linear polarization providing a polarization rotation angle in the range $0^{\circ }$–$90^{\circ }$ for the transmitted wave. Besides, the effect of twist angle $\psi =30^{\circ }$ on the polarization states manifests itself in the appearance of the points on the Poincaré sphere denoting almost circular polarization for the reflected waves. Furthermore, we have investigated the frequency evaluation of the output polarization states on Poincaré sphere for better visualization of these results. (see the supplementary information).

## Conclusion

In conclusion, we have investigated the optical response of a single and double $\textrm{WTe}_2$ thin film on a silicon substrate. We found that the transmittance and reflectance of these structures reveal crucial dependence on the polarization angle and frequency of the incident wave. These effects originate from the inherent anisotropy of the $\textrm{WTe}_2$ thin film in its $T_d$ phase that leads to difference in the permittivities along the principal axes of the thin film. Moreover, we demonstrated that the influence of the frequency of the incident wave on the optical response of these structures is more prominent in the hyperbolic regime, where thin film of $\textrm{WTe}_2$ exhibits metallic feature along one in-plain principal axis and behaves as a dielectric along the other principal axis. Furthermore, we analyzed the polarization state of the transmitted and reflected waves for a normal incident wave on these structures with an initial linear polarization. We explained that the twisted double thin film structures provide the ability for the tunable polarization rotation in the range of $0^{\circ }$–$90^{\circ }$ for the outgoing waves by adjusting the frequency and the polarization angle of the incident wave. In particular, we elucidated that altering the twisting angle of the double $\textrm{WTe}_2$ thin films yields higher level of tunability of the polarization state of the outgoing waves. The proposed structures possessing tunable polarization for transmitted and reflected waves can be utilized for applications in communication, imaging, and information processing.

### Supplementary Information

Supplementary Information.

## Data Availability

All data generated or analysed during this study are included in this published article and its supplementary information files.
