# Supplementary material for: Polarization dependent light propagation in WTe2 multilayer structure
Source: Sci Rep. 2023 Aug 14;13:13169. doi: 10.1038/s41598-023-40460-7 (PMC10425423; doi:10.1038/s41598-023-40460-7)
Supplement: Supplementary file 1 — Supplementary Information. [file 41598_2023_40460_MOESM1_ESM.pdf]

Supplementary Information for Polarization dependent light propagation in WTe_2 multilayer structure

S. Oskoui Abdol[a,b], S. Shojaei[a,b,*], B. Abdollahipour[a]

[a]*Faculty of physics, University of Tabriz, Tabriz 51666-16471, Iran*
[b]*Research Institute for Applied Physics and Astronomy (RIAPA), University of Tabriz, 51655-163, Tabriz, Iran*

1. Optical responses of single and double WTe_2 thin film

First, we present the reflectance and transmittance spectra of the bare substrate. The substrate is assumed to be a silicon layer with a thickness $d_d = 100nm$ and the refractive index $n = 3.46$, which is almost constant in the range of the wavelengthes in question $(100 - 700cm^{-1})$. As seen in Fig. 1, the spectra of the wave components show a sinusoidal dependence on the incident polarization angle, while as we expect for an isotropic medium this dependence disappears in the total spectrum. Moreover, we observe a slight frequency dependence in the total spectra which is normal for a thin layer of a dielectric. In addition, for sake of the comparison we include the spectra of the wave components and also total spectra for the single WTe_2 thin film with thickness $d = 30nm$ deposited on the Si substrate in Fig. 2. As we can see, the total spectra represents an essential dependence on the polarization angle and frequency, which reflects the inherent anisotropic and hyperbolic characteristics of the WTe_2 thin film.

Next, we present dependence of the wave propagation through a single WTe_2 thin film deposited on Si substrate on the thickness of substrate. The thickness of the WTe_2 thin film is considered to be $30nm$. The optical response of the single WTe_2 thin film as a function of frequency and thickness of substrate has

*Corresponding author
Email address: shojaei.sh@gmail.com (S. Shojaei)

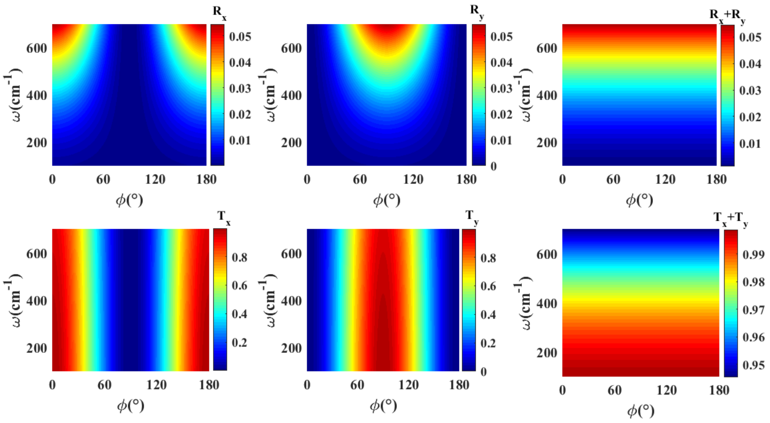

Figure 1: Plots of the reflectance and transmittance for x and y components of the electric field of the wave incident on bare substrate as a function of frequency ω and polarization angle ϕ.

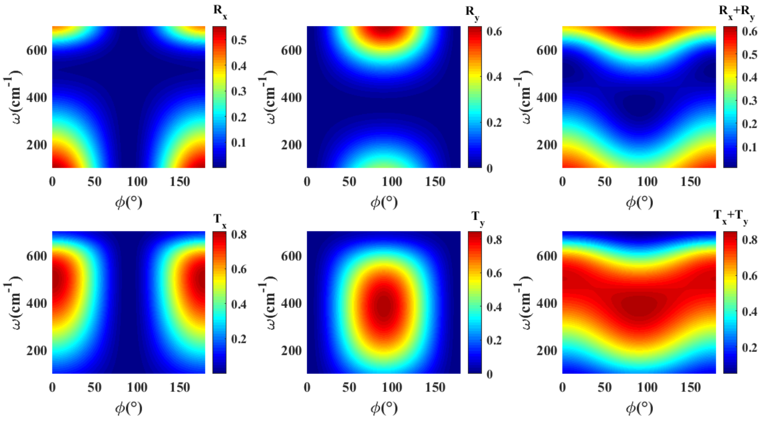

Figure 2: Plots of reflectance and transmittance for x and y components of the electric field of wave incident on the WTe_2 thin film deposited on Si substrate as a function of frequency ω and polarization angle ϕ.

been shown in Fig. 3 for incident polarization angle $\phi = 45°$. As we can see, the optical response shows considerable dependence on the thickness of the substrate. It is clear from the figures that, for a definite frequency in the hyperbolic range, increasing the thickness of the dielectric layer leads to an increase in the reflectance but a decrease in the transmittance and absorption for both x and

y directions.

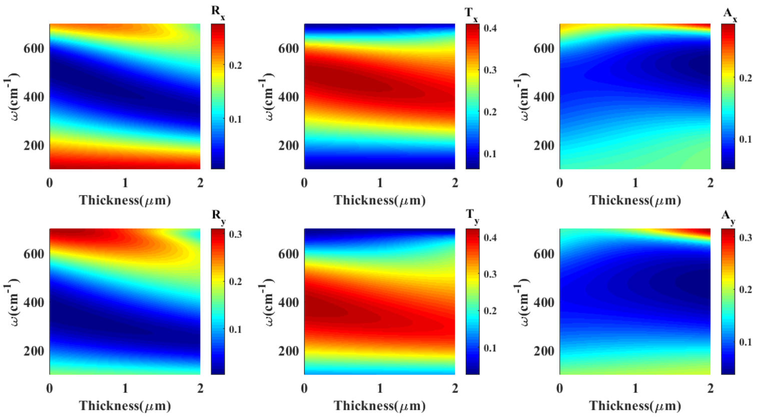

Figure 3: Plots of reflectance, transmittance and absorption for x and y components of the electric field incident on the single WTe_2 thin film as a function of frequency ω and the thickness of the dielectric layer for incident polarization angle $\phi = 45°$ and $d = 30\mu m$.

Now, we proceed to present the optical response of the untwisted double WTe_2 thin film structure with equal thicknesses of thin films $d_1 = d_2 = 30nm$ in terms of the substrate thickness. In Fig. 4 we have exhibited the dependence of the optical response on the dielectric layer thickness for incident polarization angle $\phi = 45°$. The reflectance and transmittance show considerable dependence on the thickness of the dielectric layer. In most frequencies a nearly periodic behavior is observed for both the reflectance and transmittance spectrums.

2. Polarization rotation

The inherent anisotropy of WTe_2 thin film gives rise to the change in the polarization state of the reflected and transmitted waves. In Figs. 5 and 6 the dependence of the azimuthal angle of the polarization ellipse, phase difference and the ellipticity angle on the thickness of the dielectric layer has been presented for both transmitted (up row) and reflected (down row) waves for incident polarization angle equal to $\phi = 45°$ for the single and double WTe_2

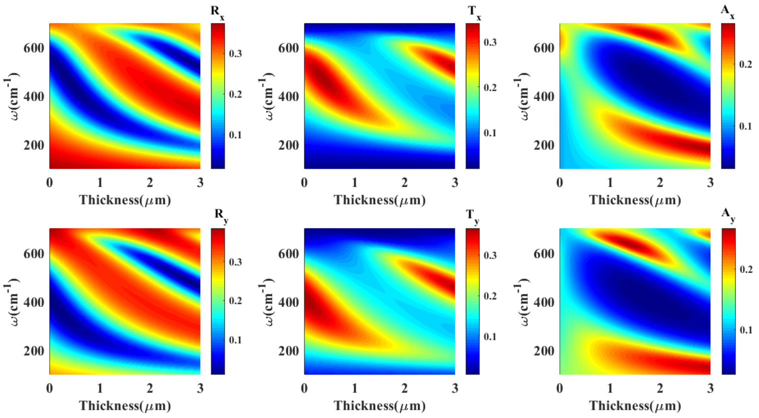

Figure 4: Plots of reflectance, transmittance and absorbance for x and y components of the electric field incident on the untwisted double WTe_2 thin film structure as a function of frequency ω and thickness of the dielectric layer for polarization angle $\phi = 45°$ and WTe_2 thin films thicknesses $d_1 = d_2 = 30nm$.

thin film structure, respectively. We observe that by adjusting the thickness of the substrate we can have 90° and 180° phase differences in a frequency region. It is obvious from these figures that frequency regions with approximately 90° and 180° phase differences move to lower frequencies and shrinks by increasing the thickness of WTe_2 thin film.

3. Polarization states on the Poincaré sphere

The polarization space (Poincaré sphere) is utilized to provide an intuitive demonstration of the output polarization states. As shown in Figure 6, each point on the surface of the sphere denotes a distinct polarization state. The normalized Stokes parameters of a polarization state as the cartesian coordinates are defined as follows,

$$\begin{aligned} S_1 &= \cos(2\eta)\cos(2\chi), \\ S_2 &= \cos(2\eta)\sin(2\chi), \\ S_3 &= \sin(2\eta), \end{aligned} \tag{1}$$

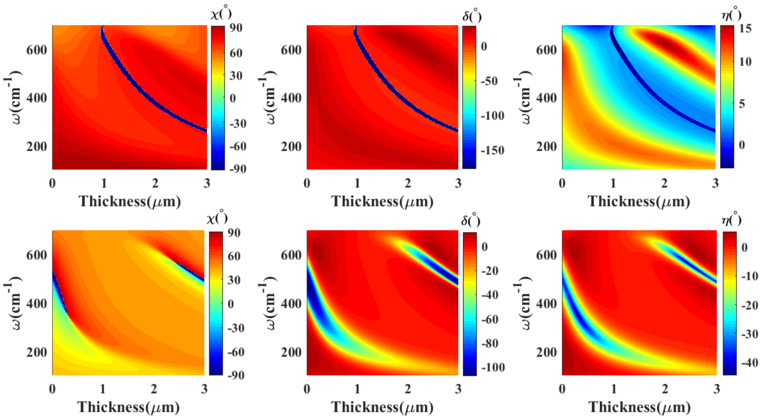

Figure 5: The azimuthal angle of the polarization ellipse (χ), phase difference (δ) and the ellipticity angle (η) as a function of frequency and thickness of the dielectric layer in the single WTe_2 thin film deposited on dielectric substrate for incident polarization angle $\phi = 45°$ for transmitted (up row) and reflected (down row) waves.

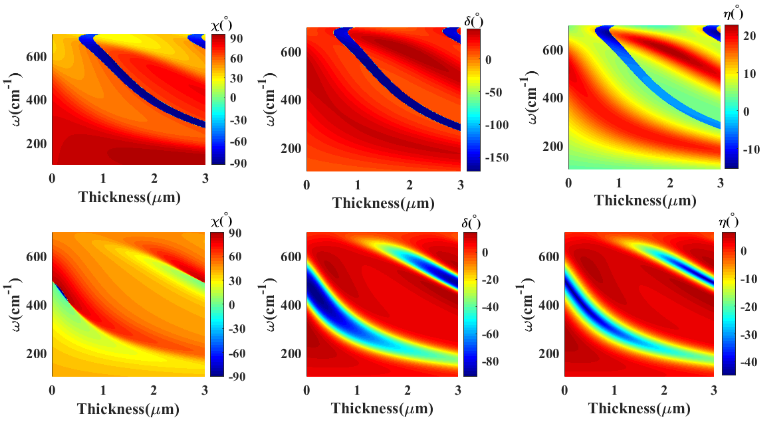

Figure 6: The azimuthal angle of the polarization ellipse (χ), phase difference (δ) and the ellipticity angle (η) as a function of frequency and thickness of the dielectric layer in untwisted double WTe_2 thin film structure for incident polarization angle $\phi = 45°$ for transmitted (up row) and reflected (down row) waves.

where χ and η are referred to as azimuth and ellipticity angles of the polarization ellipse, respectively. An arbitrary polarization state of light can be described by these angles as a point on the surface of the Poincaré sphere. Points on

the Poincaré sphere indicate distinct elliptical polarization states, except for the points on the equator corresponding to the linearly polarized states and the poles, which indicate circularly polarized states (for detail see Fig. 7).

Selected polarization state	Azimuth angle(2χ)	Ellipticity angle(2η)	Stokes parameters (S1,S2,S3)
**Horizontal Linear**	**0**	**0**	**(1,0,0)**
**+45° Linear**	**$\pi/2$**	**0**	**(0,1,0)**
**Vertical Linear**	**π**	**0**	**(-1,0,0)**
**-45° Linear**	**$3\pi/2$**	**0**	**(0,-1,0)**
**Right Circular**	**0**	**$\pi/2$**	**(0,0,1)**
**Left Circular**	**0**	**$-\pi/2$**	**(0,0,-1)**

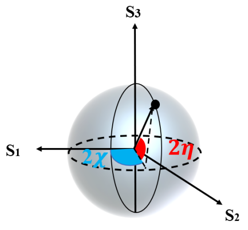

Figure 7: Presentation of distinct polarization states on the Poincaré sphere with different polarization ellipticity (2η) and azimuth angles (2χ).

The polarization states of transmitted (up row) and reflected (down row) waves exhibited on the Poincaré sphere are presented in Fig. 8 for the structures composed of the single and double WTe_2 thin film, As is seen, the polarization state of the single WTe_2 thin film indicates approximately linear polarization for the transmitted wave and represent the linear and circular polarization for the reflected wave. However, the polarization state of the double twisted WTe_2 thin film exhibits regions on the Poincaré sphere, providing a polarization rotation approximately in the range $0° - 90°$ for the transmitted wave. Moreover, the effect of twist angle $\psi = 30°$ on the polarization state appears in the approximate circular polarization for the reflected waves.

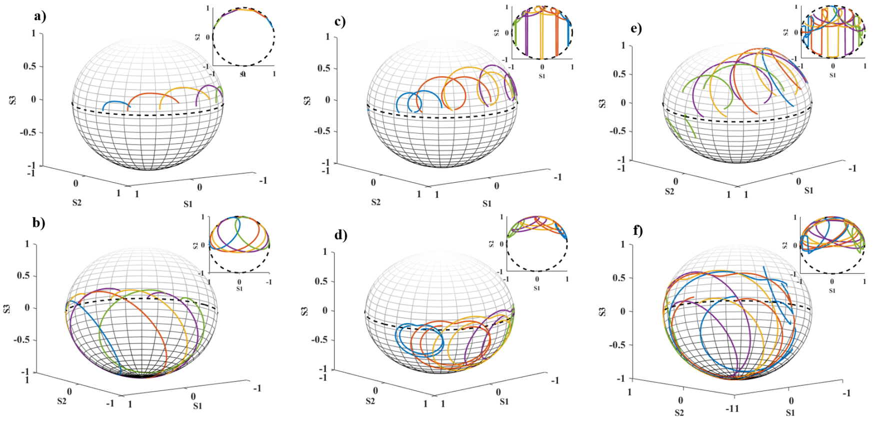

Figure 8: Polarization states on the Poincaré sphere presented for the single WTe_2 thin film in **(a,b)**, for the double WTe_2 thin film structure with $\psi = 0°$ in **(c,d)**, and for $\psi = 30°$ in **(e,f)**, for transmitted (up row) and reflected (down row) waves. The frequency evolutions of the output polarization states on the Poincaré sphere are shown for different angles of the incident polarization (ϕ), $\phi = 15°$ (blue curve), $30°$ (red curve), $45°$ (yellow curve), $60°$ (purple curve) and $75°$ (green curve). The equator of the Poincaré sphere is indicated by dashed black circle.
